# A new method for the assessment of endothelial function with peripheral arterial volume

**DOI:** 10.1186/s12872-018-0821-5

**Published:** 2018-05-04

**Authors:** Daoyuan Si, Lujia Ni, Yunfei Wang, Jinsha Liu, Jining Yang, Ping Yang

**Affiliations:** 1 Department of Cardiology, Jilin Provincial Engineering Laboratory for Endothelial Function and Genetic Diagnosis of Cardiovascular Disease, China-Japan Union Hospital of Jilin University, Xiantai Street NO.126, Changchun, Jilin China; 2 Department of Ultrasonography, China-Japan Union Hospital of Jilin University, Changchun, Jilin China

**Keywords:** Noninvasive measurement, Endothelial function, Peripheral arterial volume, Flow mediated dilation, Photoplethysmography

## Abstract

**Background:**

Currently, many methodological approaches have been developed to assess peripheral endothelial function. However, a development of the noninvasive and automated technique for routinely assessing endothelial function is still required. We evaluated the potential value of a new method to measure peripheral endothelial function with reactive hyperemia peripheral arterial volume (RH-PAV) in patients with chest pain.

**Methods:**

We used a novel oximeter-like probe to detect the peripheral arterial volume (PAV) of the finger and compared it with brachial flow-mediated dilation (FMD) performed in 93 consecutive patients with chest pain. The RH-PAV index was defined as the ratio of the digital pulse volume during reactive hyperemia relative to the baseline.

**Results:**

Ninety-three patients (53 men, 58 ± 5 years) completed the study, and 53 patients demonstrated coronary artery disease (CAD) following scheduled coronary angiography. There was a moderate linear relationship between PAV and FMD (*r* = 0.69, *p* < 0.01). Similar to FMD, PAV was more impaired in patients who have more cardiovascular risk factors (CRFs). The subjects with CAD had lower PAV and FMD, compared with those without CAD (1.05 ± 0.23 VS. 1.41 ± 0.37, *p* < 0.01; 6.7% ± 2.9% VS. 10.4% ± 2.9%, *p* < 0.01, respectively), and the relationships between FMD and PAV were also significant in both CAD (*r* = 0.54, *p* < 0.01) and non-CAD (*r* = 0.62, *p* < 0.01) patients.

**Conclusions:**

Endothelial function of digital artery assessed with the novel PAV method demonstrated a profile similar to that of brachial artery measured with FMD. The hyperemia PAV was decreased by factors which were considered to impair endothelial function, suggesting that PAV has the potential to be a novel method to study endothelial function.

## Background

Cardiovascular diseases have become a global challenge in public health, and atherosclerosis is a major one that can lead to ischemia of the heart, brain or extremities, occasionally resulting in infarction [1]. Endothelial dysfunction is an integral part to each phase of atherosclerosis, from initiation to subsequent cardiovascular complications, but can be reversible at every stage, indicating early detection of endothelial dysfunction may have therapeutic and prognostic implications [2]. Therefore, the evaluation of the endothelial function is essential for investigating the pathophysiology of cardiovascular diseases, including coronary artery disease (CAD).

Endothelial function can be evaluated by detecting its capacity to perform the various functions, such as regulation of vasomotor tone which has become the most prevailing study endpoint [3, 4]. The invasive assessment of endothelial function by catheterization was thought to be the “gold standard” in initial studies, but the invasive and costly nature prohibit its clinical utility [5]. Later on, flow mediated dilation (FMD), a noninvasive method of investigating peripheral artery, became widely used in the last few decades [6]. However, there were still several methodological challenges might limit its application in daily practice, such as being operator-dependent and systemic changes during testing [7]. Then peripheral arterial tonometry (PAT) gained increasing attention, based on similar physiological mechanisms as FMD, including the measurement of vasodilation in response to reactive hyperemia. Although growing evidence indicates that the promising technique potentially impacts the field of cardiovascular study, a number of methodological and clinical aspects still need to be clarified [8, 9].

Nonetheless, the development of a noninvasive and automated technique for routinely assessing endothelial function is still required, from a clinical perspective. The measurement of pulsatile volume changes of index finger during reactive hyperemia has been proved to be an effective way to evaluate endothelial function [10]. Forearm plethysmography was also shown to be a useful method to assess endothelial function [11, 12]. So we used a novel oximeter-like finger probe (peripheral arterial volume [PAV]) to measure the changes of pulsatile blood flow volume during reactive hyperemia in our study, detecting the changes of hemoglobin (Hb) flow. Our aim was to demonstrate the relationship between FMD and PAV in patients with chest pain.

## Methods

### Study objective

Patients were eligible for enrollment if they had chest pain, were scheduled to undergo coronary angiography (CAG) and were aged between 30 to 75 years. Exclusion criteria included acute coronary syndrome; left ventricular ejection fraction < 55%; Raynaud’s disease; atrial fibrillation; valvular heart disease; chronic respiratory; renal disease; thyroid disorders or sex hormone disorders. This study protocol conformed to the ethical guidelines of the Helsinki Declaration, approved by the ethical review board of China-Japan Union Hospital of Jilin University (2017040603), and the written informed consents were signed by all patients.

The following cardiovascular risk factors (CRFs) were evaluated in each individual: male sex; hypertension(systolic blood pressure > 140 mmHg or/and diastolic blood pressure > 90 mmHg, or under antihypertensive treatment); diabetes mellitus (fasting glucose levels > 126 mg/dL, or under antihyperglycemic treatment); hyperlipidemia (total serum cholesterol level > 200 mg/dL or taking lipid-lowering treatment); family history of CAD; postmenopause; and current smoking(have smoked 100 cigarettes in their lifetime and currently smoke cigarettes).

In a dimly lit and temperature controlled room, PAV and FMD were performed on the early morning of the scheduled CAG. Participants were instructed to refrain from vasoactive medication, food, caffeine, tea, tobacco, or exercise for at least l2 hours before the measurement. CAD was defined as 50% or more luminal narrowing in one or more major epicardial vessels as determined by CAG.

### FMD testing

FMD was measured according to the guidelines [13]. Following a 10-min equilibrium period, brachial artery diameter was assessed at baseline and post reactive hyperemia with a 12-MHz linear-array peripheral vascular probe (Philips iE33 ultrasound system). The transient ischemia was induced by inflating a forearm blood pressure cuff to 50 mmHg above patient’s resting systolic blood pressure for 5 min; then brachial artery diameter was measured at 1, 2 and 3 min post cuff deflation. Percent change in flow-mediated dilation (%FMD) was defined as the maximal brachial artery diameter due to reactive hyperemia compared to the baseline diameter. Percent change in nitroglycerin-mediated dilation (%NMD) was defined as the maximal brachial artery diameter during a 3 min period following administration of nitroglycerin, compared with the baseline diameter. All measurements were performed twice by two independent observers who were blinded to the patients’ information.

### PAV testing

The basic principle of PAV is the same as FMD and PAT, this technique (Saintyear Medical Ltd., Shenzhen, China) includes a photo-plethysmographically based index finger probe to measure digital arterial volume changes accompanying pulse waves. Light-emitting diodes (940 nm) in conjunction with a light-sensitive sensor were used to assess the absorption of the infrared light. The calculation was made by the difference in digital artery hemoglobin flow between pre- and post-occlusion of an upper limb.

**Fig. 1:**
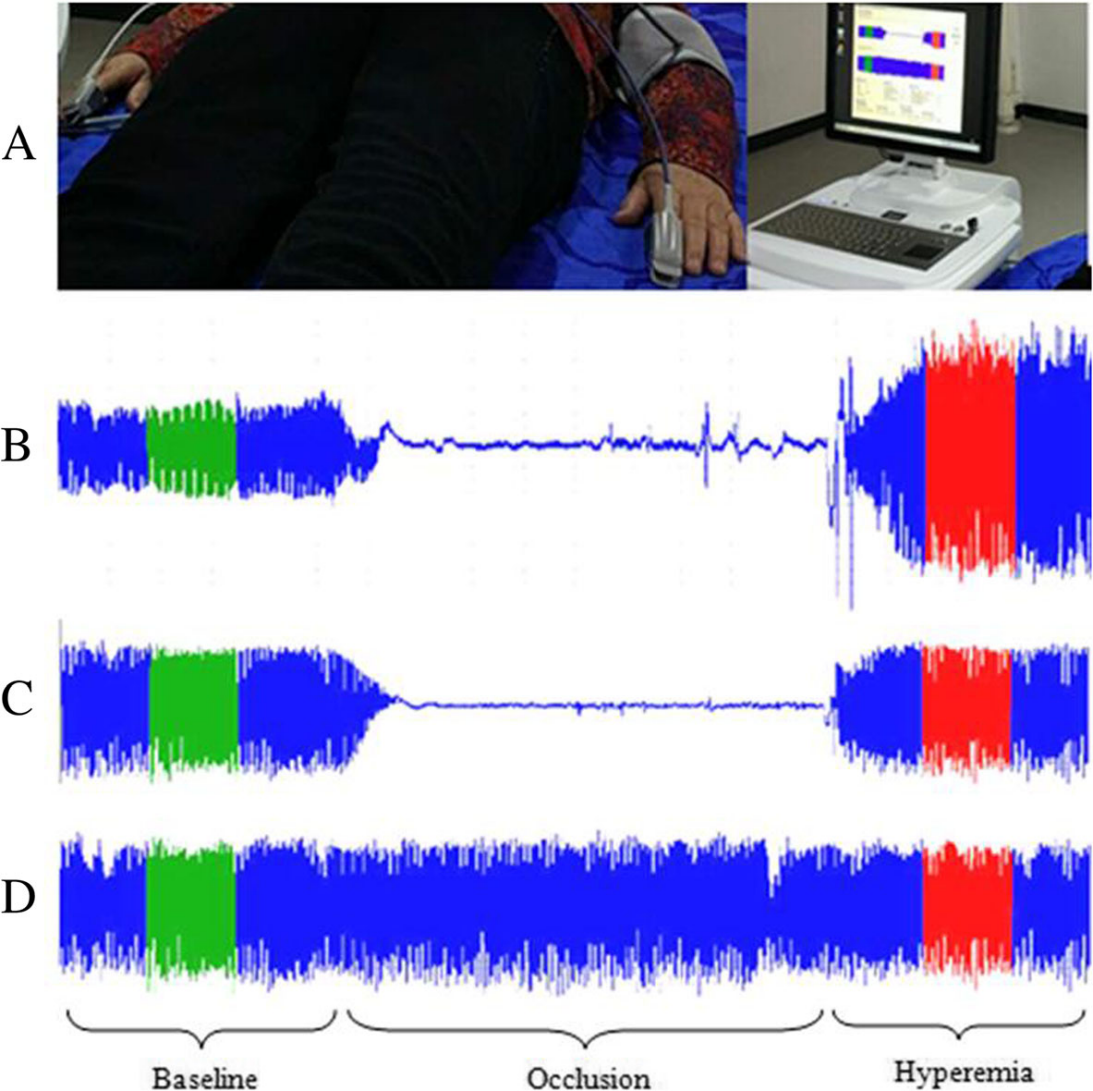
**a** setup of PAV testing, **b** and **c**, from the side undergoing hyperemia. **d** from the contralateral side. **b** Individual with non-CAD and fewer CRFs showing an increased PAV signal during hyperemia. C, Individual with CAD and more CRFs showing a blunted PAV response during hyperemia. D, PAV recording from the same patient with CAD and more CRFs in contralateral side

The blood pressure cuff was placed on the left upper arm undergoing hyperemia testing, while the contralateral arm served as a control. PAV was performed by placing the probes on the index finger of each (Fig. 1). The occlusion was induced by inflating the cuff to 50 mmHg above patient’s resting systolic blood pressure for 5 min. Reactive hyperemia PAV was defined as the ratio of the average pulse wave amplitude (PWA) during the 40-s period beginning after exactly 40 s of reactive hyperemia compared with the average PWA during the 40-s period beginning after exactly 40 s of pre-occlusion baseline. The result of PAV was calculated automatically normalizing to the contralateral arm to correct for any systemic changes during the test. The nitroglycerin response (PAV nitroglycerin) was calculated as the ratio of the average PWA during the 40-s period beginning exactly after 3 min after administration of nitroglycerin compared with the average PWA during the 40-s period beginning after exactly 40 s of the second baseline, and no contralateral correction was applied.

### Statistical analysis

Data are presented as mean ± standard deviation(SD). The two-sided t-test, Pearson rank correlation, and linear regression analysis were used to assess the relationship between FMD and PAV. Univariate analysis was used to evaluate the association between the PAV and various CRFs. Receiver operating characteristic (ROS) curves of FMD and PAV was performed to assess the discrimination performance of CAD. All analyses were done by SPSS 17.0 statistical software (SPSS Inc., Chicago, Illinois). *P* ≤ 0.05 was considered statistically significant.

## Results

### Study population

Ninety-three individuals (58 ± 5 years) completed the study, and 53 of them had CAD. We describe the characteristics of our study population through Table 1. Left ventricular ejection fraction (62% ± 2%) was normal in each patient, and approximately half of the patients had hypertension. 23% of subjects were reported a family history of CAD, 24% of subjects had diabetes mellitus, and 70% of subjects had hyperlipidemia.

**Table 1:** Study population characteristics

Variable	*n* = 93
Age(years)	58 ± 5
Male, n(%)	53(57)
Body mass index(kg/m^2^)	24.6 ± 3.3
Left ventricular ejection fraction(%)	62 ± 3
Coronary artery disease	53(57)
Systemic hypertension(%)	59(53)
Diabetes mellitus(%)	22(24)
Hyperlipidemia(%)	65(70)
Current smoking(%)	54(58)
Family history of coronary disease(%)	21(23)
Postmenopause(% of women)	30(75)

Values are presented as mean ± SD or number (%)

### Relation between FMD and PAV

**Fig. 2:**
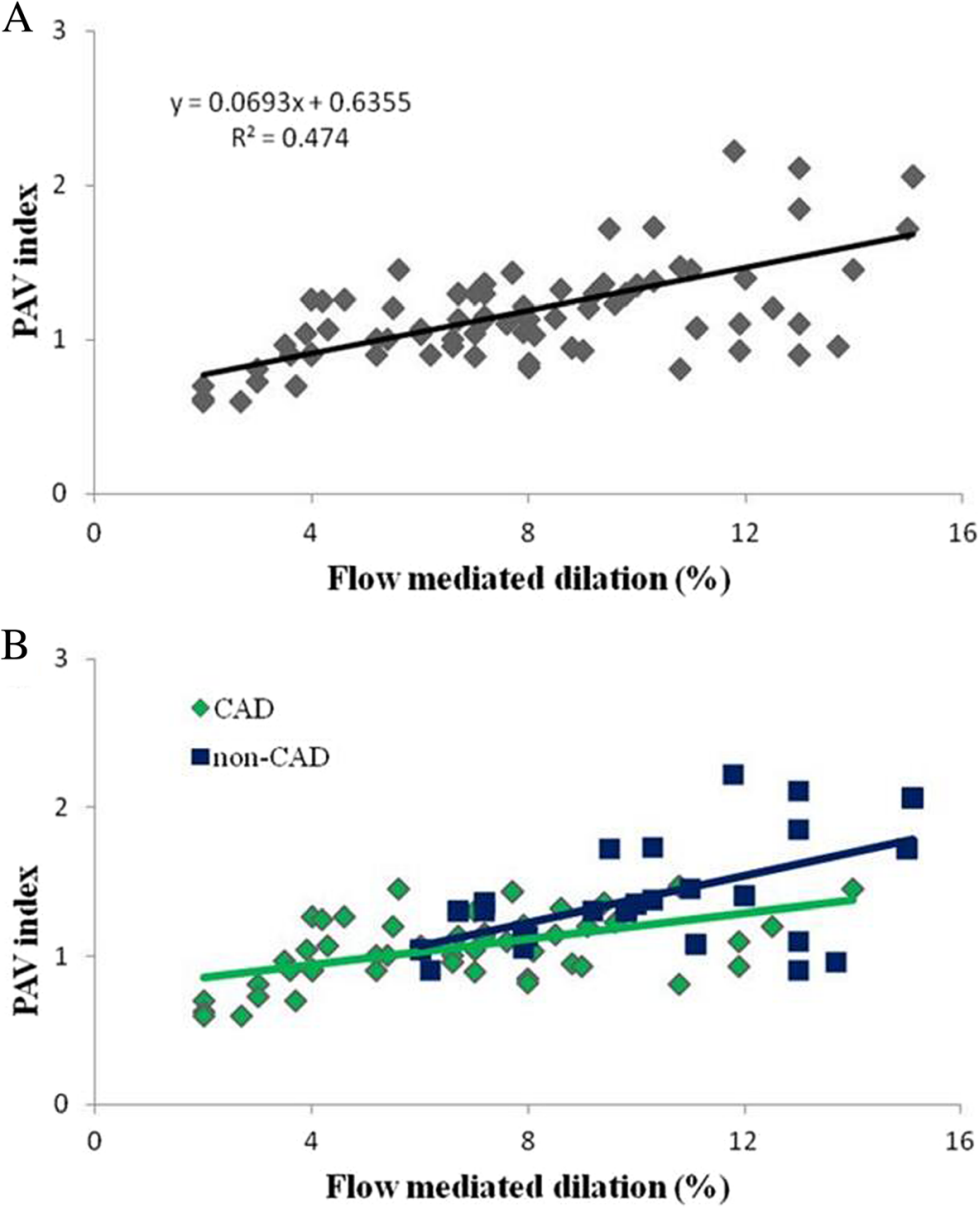
**a** Linear regression evaluation of the relation between PAV index and FMD of the brachial artery in all patients (*r* = 0.69, *P* < 0.01). **b** Linear regression evaluation of relation between PAV index and FMD in CAD(*r* = 0.54, *P* < 0.01, *n* = 53) and non-CAD (*r* = 0.62, *P* < 0.01, *n* = 40) population

The average of FMD and PAV were 8.3% ± 3.4%(range 2.0-15.1%) and 1.21 ± 0.35(range 0.60-2.22), respectively. Linear regression analysis revealed that FMD and PAV were significantly correlated in all patients (*r* = 0.69, *p* < 0.01) (Fig. 2a). The significant relationships were also identified between FMD and PAV in both CAD (*r* = 0.54, *p* < 0.01) and non-CAD (*r* = 0.62, *p* < 0.01) populations (Fig. 2b). The average of NMD was 13.9% ± 4.3% (range 5.1-26%), whereas the average of nitroglycerin-PAV was 1.52 ± 0.37 (range 0.81-2.7, *r* = 0.65, *p* < 0.01).

### Relation between CRFs and PAV

**Fig. 3:**
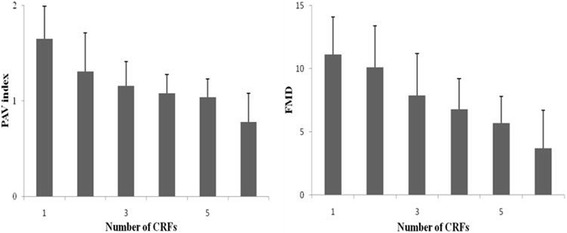
Similar to brachial artery FMD, the PAV index generally correlated with the numbers of CRFs (*r* = 0.53, *P* < 0.01 for FMD, *r* = 0.45, *P* < 0.01 for PAV). Values are expressed as the mean ± SD

The average number of CRFs was 3.0 ± 1.2 in each patient. Spearman correlation analysis revealed a significant relationship between the number of CRFs and PAV (*r* = 0.45, *p* < 0.01), as well as FMD (*r* = 0.53, *p* < 0.01), and the trend is similar (Fig. 3). Eight patients with < 2 CRFs had the PAV of 1.65 ± 0.36, 51 patients with 2 to 3 CRFs had the PAV of 1.25 ± 0.35, and 34 patients with > 3 CRFs had the PAV of 1.04 ± 0.21 (< 2 vs. 2-3 CRFs *P* < 0.05, 2-3 vs. > 3 CRFs *P* < 0.01, < 2 vs. > 3 CRFs *P* < 0.01). The PAV was significantly lower in patients with hypertension(1.10 ± 0.31), hyperlipidemia(1.11 ± 0.31), diabetes mellitus(1.02 ± 0.23) or family history of CAD(1.17 ± 0.25) than those without such CRF(1.32 ± 0.35 *P* < 0.01, 1.30 ± 0.35 *P* < 0.01, 1.26 ± 0.36 *P* < 0.01, 1.22 ± 0.37, respectively). Postmenopausal women (1.13 ± 0.34) had lower PAV than premenopausal ones(1.5 ± 0.32 *P* < 0.01). There was no significant relationship between the PAV and the gender, age, or current smoking.

### Relation between CAD and PAV

**Fig. 4:**
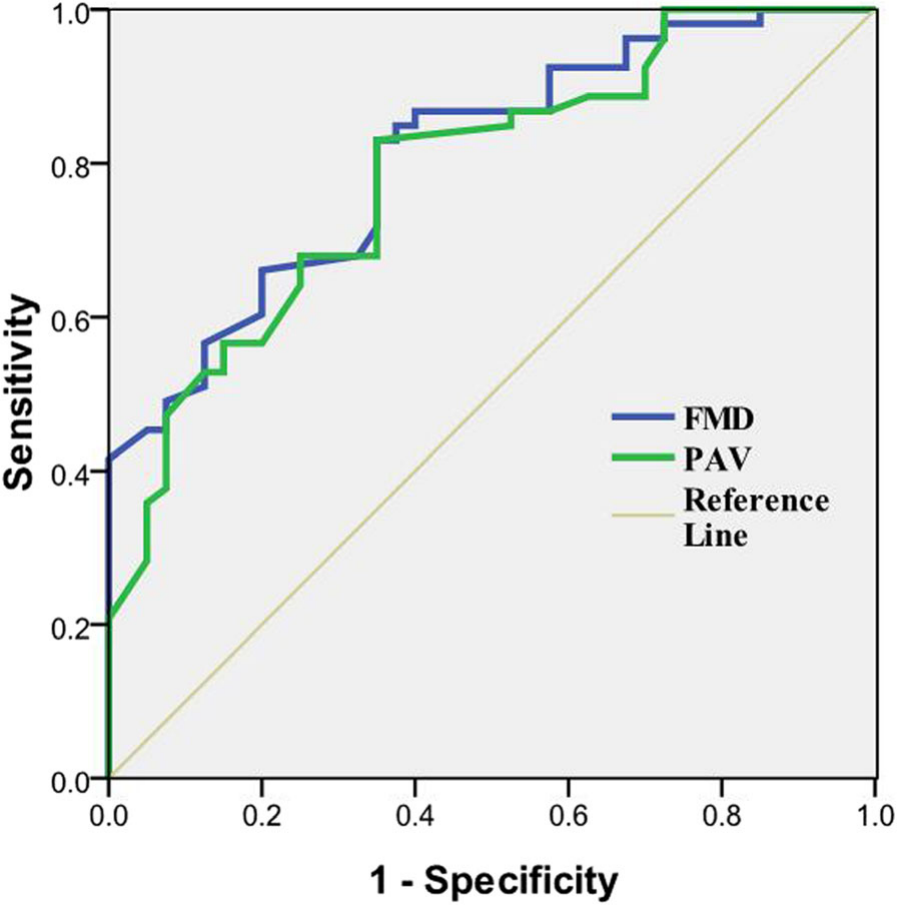
ROCs of FMD and PAV for the prediction of coronary heart disease

The subjects with CAD had lower PAV(1.05 ± 0.23) and FMD (6.7 ± 2.9%) compared with those without CAD (1.41 ± 0.37, 10.4 ± 2.9%, *P* < 0.01 for both). The area under the curve (AUC) of PAV and FMD was 0.785 (95%CI 0.694-0.877, *P* < 0.01) and 0.810 (95%CI 0.725-0.895, *P* < 0.01) for the prediction of CAD in ROC curves (Fig. 4). There was no significant difference between the AUCs of PAV and FMD (Z = 0.54 < 1.96, *P* > 0.05) with the pairwise comparison of ROC curve test. The sensitivity and specificity of PAV were 83 and 65%, while those of FMD were 85 and 63% for detection of CAD when 1.28 of PAV and 9.8% of FMD was identified as the cutoff points.

## Discussion

Our pilot study was performed to evaluate the feasibility of reactive hyperemia PAV to assess endothelial function of the peripheral artery in patients with chest pain. The data demonstrated that the significant relationship between PAV hyperemia ratio and brachial artery FMD not only existed in all subjects but also in both CAD and non-CAD groups. Similar to FMD, peripheral arterial reactivity, defined as the PAV, also correlated with traditional CRFs and CAD. Since there is a significant relationship between abnormalities in the central and peripheral arteries in the process of atherosclerosis, peripheral vascular endothelial function testing has been evolving to be more feasible for daily test and screening large numbers of people [2]. Our findings were consistent with prior studies of peripheral vascular endothelial function testing [14, 15]. The PAV technique could represent a new method to assess endothelial function from peripheral vascular beds, contributing to the development of noninvasive measurement of endothelial function. Although the brachial and digital measurements of vascular function may reveal distinct information and reflect different characterizations of a disease stage, the association of both was observed in aged patients or who have more advanced CRFs in prior studies [16, 17].

The PAV was lower in patients with diabetes mellitus, hypertension, hyperlipidemia, or family history of CAD, but there was no significant relationship between the decreased PAV and age, gender or current smoking. Previous studies have reported that the prevalence of reduced FMD increased substantially with advancing age, and the association attributed to alterations in the production of nitric oxide and oxidant species [17, 18]. In contrast, low PAV showed little variability with advancing age in our study. Actually increasing age also did not associate with abnormal PAT in several studies [16, 19]. The findings may potentially be explained by the counterbalance physiologic changes in the finger microvessels with aging that tend to preserve the reactive hyperemia [15, 16]. Recently Ulrike et al. [20] determined that PAT ratio increased significantly with increasing age in 445 children and adolescents, so it was also possible because of a large number of old patients who were enrolled in our study. Men always had lower values for noninvasive measurements of endothelial function, such as FMD, in prior reports [15, 17, 21, 22], but sex was not found to affect PAV ratio in our study. The possible explanation might be the high rate of postmenopausal women (75%) enrolled in the present study. The enrollment bias might be the reason why current smoking had no significant effect on PAV in the study.

Our study demonstrated that patients with CAD had attenuated PAV, compared to those without CAD, and the discrimination value of CAD existence was similar between PAV and FMD. Although it has been reported that peripheral vascular endothelial function measured by blood flow or reactive hyperemia after cuff release are not solely nitro oxygen-mediated, it possesses independent predictive value [16]. Vasodilation measurements of conduit artery and microarray probably reflect a distinct aspect of vascular function complementarily [2, 16]. Our findings consisted of previous studies about other measurements of endothelial function including FMD and PAT, suggesting that assessment of peripheral endothelial function using PAV could be potentially valuable for identifying patients with CAD [14, 23, 24].

### PAV technique

The absence of major muscle, high vascular density and good perfusion of the finger make it accessible for measuring changes in the volume of blood flow [25]. The photoplethysmographic recordings of PAV were derived from the index finger in the study, and the similar technique was also used in the analysis of digital volume pulse (DVP) and pulse oximetry [26, 27]. Technically speaking, PAV is likely to be the surrogate for arterial distensibility of the vascular digital district, as the blood volume in the veins and capillaries, as well as the bone, fat, and skin, remain relatively constant while the blood volume in the artery increases during systole and decreases during diastole. According to the Beer-Lambert Law in a modeled blood vessel, simply measuring the absolute absorbance in the finger would be an inaccurate estimate of arterial volume since distended distal venous blood volume would also contribute to the value measured. Nevertheless, the PAV system is able to use similar mathematics which is also used in the pulse oximeter to extract only arterial absorbance of Hb from the total signal by measuring changes in absorbance over time [26]. The influence of these relatively stationary facts, such as distended distal venous caused by occlusion, can be excluded from the automatical calculation of PAV. So instead of thimble-shaped pneumatic finger probes, used in PAT test and be considered vulnerable and disposable [28], reusable finger probes are used in PAV technique. That may potentially contribute to the daily use in the future.

### Limitations and future directions

As described above, the PAV technique is operator independent, analyzing automatically, requiring less training and the contralateral arm serves as its internal control that can be used to correct for any systemic changes during the test. Several limitations of this study and the technique deserve to be mentioned. First, only traditional CRFs were considered and other risk factors may also have potential to impair endothelial function. Gender, age and current smoking were acknowledged to impair endothelial function, but the relationships between them and PAV were not significant, the bias may affect the result. Second, reproducibility is important when vascular reactivity tests are compared [29]. Future studies should investigate the variation of PAV and explore the pathophysiology of PAV, including the anatomical and hemodynamic determinants. Third, Due to the different absorbance of oxygenated and deoxygenated hemoglobin in 940 nm wavelength infrared light, the calculation of PAV that takes total hemoglobin into account may be inaccurate in patients with low oxygen saturation. So chronic respiratory disease was included in the exclusion criteria of our study. 800 nm wavelength probably should be studied, where the absorbance of oxygenated and deoxygenated hemoglobin are equal, as well as an improvement strategy. Finally, the population enrolled was relatively small and all subjects underwent coronary angiography for clinically suspected CAD. Therefore, selection bias may affect the results, larger cohorts are needed for further study.

## Conclusion

Our data indicated that the novel PAV method showed similar patterns of abnormality to FMD in patients with chest pain. PAV is influenced by factors proven to affect endothelial function, such as the presence of CRF and CAD. In addition, PAV is potentially useful for identifying patients at high risk for CAD, suggesting that PAV has the potential to be a novel noninvasive method in the field of cardiovascular research.

## Abbreviations

AUC: Area under curve
CAD: Coronary artery disease
CAG: Coronary angiography
CRF: Cardiovascular risk factor
FMD: Flow mediated dilation
Hb: Hemoglobin
NMD: Nitroglycerin mediated dilation
PAT: Peripheral arterial tonometry
PAV: Peripheral arterial volume
PWA: Pulse wave amplitude
ROC: Receiver operating characteristic curve
SD: Standard deviation
